# Bacterial diversity analysis of larvae and adult midgut microflora using culture-dependent and culture-independent methods in lab-reared and field-collected *Anopheles stephensi*-an Asian malarial vector

**DOI:** 10.1186/1471-2180-9-96

**Published:** 2009-05-19

**Authors:** Asha Rani, Anil Sharma, Raman Rajagopal, Tridibesh Adak, Raj K Bhatnagar

**Affiliations:** 1 Insect Resistance Group, International Centre for Genetic Engineering and Biotechnology (ICGEB), ICGEB Campus, Aruna Asaf Ali Marg, New Delhi, 110 067, India; 2 National Institute of Malaria Research (ICMR), Sector 8, Dwarka, Delhi, 110077, India

## Abstract

**Background:**

Mosquitoes are intermediate hosts for numerous disease causing organisms. Vector control is one of the most investigated strategy for the suppression of mosquito-borne diseases. *Anopheles stephensi *is one of the vectors of malaria parasite *Plasmodium vivax*. The parasite undergoes major developmental and maturation steps within the mosquito midgut and little is known about *Anopheles*-associated midgut microbiota. Identification and characterization of the mosquito midgut flora is likely to contribute towards better understanding of mosquito biology including longevity, reproduction and mosquito-pathogen interactions that are important to evolve strategies for vector control mechanisms.

**Results:**

Lab-reared and field-collected *A. stephensi *male, female and larvae were screened by "culture-dependent and culture-independent" methods. Five 16S rRNA gene library were constructed form lab and field-caught *A. stephensi *mosquitoes and a total of 115 culturable isolates from both samples were analyzed further. Altogether, 68 genera were identified from midgut of adult and larval *A. stephensi*, 53 from field-caught and 15 from lab-reared mosquitoes. A total of 171 and 44 distinct phylotypes having 85 to 99% similarity with the closest database matches were detected among field and lab-reared *A. stephensi *midgut, respectively. These OTUs had a Shannon diversity index value of 1.74–2.14 for lab-reared and in the range of 2.75–3.49 for field-caught *A. stephensi *mosquitoes. The high species evenness values of 0.93 to 0.99 in field-collected adult and larvae midgut flora indicated the vastness of microbial diversity retrieved by these approaches. The dominant bacteria in field-caught adult male *A. stephensi *were uncultured *Paenibacillaceae *while in female and in larvae it was *Serratia marcescens*, on the other hand in lab-reared mosquitoes, *Serratia marcescens *and *Cryseobacterium meninqosepticum *bacteria were found to be abundant.

**Conclusion:**

More than fifty percent of the phylotypes were related to uncultured class of bacteria. Interestingly, several of the bacteria identified are related to the known symbionts in other insects. Few of the isolates identified in our study are found to be novel species within the gammaproteobacteria which could not be phylogenetically placed within known classes. To the best of our knowledge, this is the first attempt to study the midgut microbiota of *A. stephensi *from lab-reared and field-collected adult and larvae using "culture-dependent and independent methods".

## Background

Mosquitoes are transmitters of several serious human diseases including malaria. *Anophelines *are the only transmitters of malaria. *Anopheles stephensi *is the main vector in urban India, where 70% of world-wide malaria related cases occur. During the development and maturation of parasite in vector the midgut of the female *Anopheles *is a major site of interaction. Interruption of parasite development in mosquitoes remains the enticing strategy for the control of mosquito-borne diseases. The malaria parasite development involves critical steps within the mosquito midgut, an environment it shares with gut-residing bacteria. The occurrence of apparent 'symbiotic' association between *Anopheles *mosquitoes and bacterial species has not been much evaluated.

A possible approach to restrict malaria parasite transmission is to manipulate the mosquito functional genome, one possible approach is to employ normal bacterial symbionts of the mosquito gut to block development cycle in the vector. Gut microbes have been described to be involved in supporting normal growth and development of *Drosophila*. There have been conflicting reports regarding the role of microbes in the fitness of the vector. Hedges *et al*. (2008) described that *Drosophila melanogaster *flies infected with a common bacterial endosymbiont, *Wolbachia *display reduced mortality induced by a range of RNA viruses and bacterial presence provides a fitness advantage to flies. The study highlighted the notion that the native microbes are symbionts that modulate immune responses [1]. On the other hand, *Wolbachia pipientis *wMelPop strain presence in dengue vector *Aedes aegypti*, reduced the life span of vector to half the normal adult life span. Nevertheless, it is becoming abundantly clear that endosymbiont microbes have a profound influence on the vector persistence and competence in nature [2].

Mosquito midgut is an immune-competent organ. *Plasmodium *presence in gut is known to induce immune responses elsewhere in body, probably due to immune-signaling [3,4]. The intensively investigated question is whether mosquito midgut resident endosymbiont contribute towards elicitation of immune response of host to *Plasmodium *invasion? If they do indeed contribute towards facilitation of *Plasmodium *development in mosquito, the second important question is can these endosymbionts be used as paratransgenic to block their development? It is coceivable that a vector endosymbiont may be manipulated to produce antiparasitic molecules. This vector could then reintroduced into the insect gut, thus inhibiting parasite development [5-7]. A close relationship between gut microflora and mosquito development is exemplified during the metamorphosis of larva into adult mosquito. During metamorphic transition from larvae to adult the microflora associated with larvae is 'cleaned' and adult mosquitoes acquire new set of microbes. This process of microbial cleansing and acquisition is termed as gut-sterilization [8].

A few studies have been performed to identify bacterial species in field-collected *Anopheles *mosquitoes, using microbe culturing techniques. These studies highlighted breadth of bacterial flora associated with mosquitoes. Bacteria, *Pseudomonas cepacia, Enterobacter agglomerans*, and *Flavobacterium *spp. were found in high abundance in laboratory-reared *A. stephensi, A. gambiae *and *A. albimanus *mosquitoes [9]. Further, the gut microflora varied depending upon the ecological niche or geographical location of the mosquitoes. Straif *et al*. (1998) identified *Pantoea agglomerans *(synonym *Enterobacter agglomerans*) and *Escherichia coli *as the most frequently isolated bacteria, from midgut of *A. gambiae *and *A. funestus *mosquitoes caught in Kenya and Mali [10]. Jadin *et al*. (1966) identified *Pseudomonas *sp. in the midgut of mosquitoes from the Democratic Republic of the Congo [11]. Gonzalez-Ceron *et al*. (2003) isolated various *Enterobacter *and *Serratia *sp. from *Anopheles albimanus *mosquitoes captured in southern Mexico [12]. Recently, field-captured *A. gambiae *mosquitoes in a Kenyan village were reported to consistently associate with a *Thorsellia anophelis *lineage that was also detected in the surface microlayer of rice paddies [13]. The microbial flora associated with *Anopheles darlingi*, a major Neotropical malaria vector, was found to be closely related to other vector mosquitoes, including *Aeromonas*, *Pantoea *and *Pseudomonas *species. Laboratory-reared *A. stephensi *has been reported to stably associate with bacteria of the genus *Asaia *[14]. The successful colonization of *Serratia marcescens *in laboratory-bred *A. stephensi *has also been established [15].

However, it should be emphasized that microbial studies of the midgut of *Anopheles *are scarce, and have depended mainly on traditional culture-based techniques [9,10,12]. In *A. gambiae*, few studies have combined culture and PCR-based approaches to characterize gut associated bacteria [16]. Therefore, the application of "culture-dependent and culture- independent" based tools, such as 16S rRNA gene sequencing and metagenomics, to study these systems are highly desirable. 16S rRNA gene sequencing and metagenomics, have been primarily responsible in revealing the status of our lack of knowledge of microbial world such that half of the bacterial phyla recognized so far consist largely of these as yet uncultured bacteria [17]. It also provides, an idea of species richness (number of 16S rRNA gene fragments from a sample) and relative abundance (structure or evenness), which reflect relative pressure that shape diversity within biological communities [18].

There is current interest in the use of microorganisms as biological control agents of vector-borne diseases [19-21]. Microorganisms associated with vectors could exert a direct pathogenic effect on the host by interfering with its reproduction or reduce vector competence [22-25]. In laboratory-raised insects, the bacteria in the midgut can be acquired both transstadially and through contaminated sugar solutions and bloodmeals. In wild populations, however, the origin of the midgut bacteria, are still unknown [9,10,26,27]. An understanding of the microbial community structure of the mosquito midgut is necessary, which will enable us to identify the organisms that play significant roles in the maintenance of these communities. To understand the bacterial diversity and to identify bacterial candidates for a paratransgenic mosquito, we conducted a screen for midgut bacteria from lab-reared and wild-caught *A. stephensi *mosquitoes using "culture-dependent and culture-independent" approach.

## Results

### Isolation and biochemical characterization of bacterial isolates

Plating of the mosquito midgut contents from lab-reared and field-collected adult *A. stephensi *(male/female/larvae) was used for the isolation of the culturable micro flora. The bacterial colonies on TSA and LB agar were selected on the basis of minor variations using conventional microbiological techniques. The initial number of isolates was reduced based on colony characteristics (involving colony size, shape, color, margin, opacity, elevation, and consistency) and the morphology of isolates studied by Gram staining. Microbial isolates were further selected on the basis of physiological parameters such as their sensitivity to different antibiotics (see Additional file 1). It ensured the diversity of microbes at a preliminary level. The abilities of these microbial isolates to solublize the various substrates such as amylase, lipase and protease were also quite variable, few *Bacillus *strains were among the high protease producers, whereas *Enterobacter *sp. were showing high lipase activity. Overall activity in all strains was moderate, with no activity observed (zone of hydrolysis) in few of the isolates. To determine the phylogenetic relatedness of the strains, mosquito midgut contents were subjected to analysis with the 16S rRNA gene sequencing using "culture-dependent and culture-independent" approaches. Five 16S rRNA clone libraries were constructed and approximately 150 sequences per library were analyzed.

### Diversity of Cultured Bacteria from lab-reared adult *A. stephensi*

**Figure 1:**
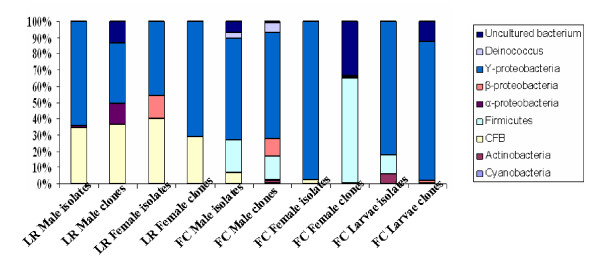
**Percentage abundance diagram of culturable isolates and 16S rRNA gene library clones from lab-reared (LR) and field-collected (FC) adult male, female and larvae of *Anopheles stephensi***. Percentage distribution was calculated on the basis of relative abundance in the total PCR amplification.

Out of a total of 50 screened bacterial colonies, 34 distinct isolates, 18 from adult male and 16 from adult female lab-reared *A. stephensi *were studied further. 16S rRNA sequencing placed these two sets of 18 and 16 isolates with their closest matches into 4 major groups. In lab- reared adult male *A. stephensi *isolates, 3 major groups were: Cytophaga-flavobacter-bacteroidetes (CFB), alphaproteobacteria and gammaproteobacteria, whereas in lab-reared adult female betaproteobacteria was also identified (Figure 1). 16S rRNA gene sequence identified the lab-reared adult male bacterial isolates as *Agrobacterium *sp., *Chryseobacterium meninqosepticum, Pseudomonas mendocina *and *Serratia marcescens*, whereas in lab-reared *A. stephensi *adult female *Comamonas *sp. was also present, the details of which are shown in Table 1. In lab-reared adult male and female *A. stephensi*, most abundant and diverse members were of gammaproteobacteria (61% and 43% respectively) particularly, *Pseudomonas mendocina *and *S. marcescens*, as a dominant group. It was followed by CFB group bacteria (*Chryseobacterium meninqosepticum*) constituting around 33% and 38% in male and female *A. stephensi*, respectively. Distinctive representative genera in lab-reared female *A. stephensi *was *Comamonas *sp. (betaproteobacterium), representing 13% of total isolates. However, male *A. stephensi *isolates were distinguishable by genera such as *Agrobacterium *sp., an alphaproteobacterium. *Chryseobacterium*, *Pseudomonas *and *Serratia *were genera common to adult male and female *A. stephensi*.

**Table 1:** Abundance of isolates and clones within the bacterial domain derived from the 16S rRNA gene sequences of lab-reared adult *A. stephensi*.

Division	Adult Male Culturable		Adult Male Unulturable		Adult Female Culturable		Adult Female Unulturable	
	
	OTU^a^	Closest database matches	OTU	Closest database matches	OUT	Closest database matches	OTU	Closest database matches
CFB group	4(6)^b^	*Chryseobacterium* *meninqosepticum*	3(8)	*C. meninqosepticum*	4(6)	*C. meninqosepticum*	2(6)	*C. meninqosepticum*

Firmicutes	-	**-**	1(1)	*Elizabethkingia* *meninqosepticum*	-	-	1(1)	*E. meninqosepticum*

Alpha proteobacteria	1(1)	*Agrobacterium* sp.	2(2)	*A. tumefaciens*	-	-	-	-

Beta proteobacteria	-	**-**	-	-	2(3)	*Comamonas *sp.	-	-

Gamma proteobacteria	3(4)	*Pseudomonas* *mendocina*	1(1)	*P. tolaasii*	2(2)	*P. mendocina*	-	-
	
	3(7)	*Serratia marcescens*	4(8)	*S. marcescens*	3(5)	*S. marcescens*	3(15)	*S. marcescens*
	
	-	**-**	1(1)	*Klebsiella *sp.	-	**-**	1(2)	*Serratia *sp.

Unclassified Bacteria	-	**-**	3(3)	Uncultured bacterium clone	**-**	**-**	**-**	**-**

Total	11 (18)	Species = 4	15 (24)	Species = 7	11 (16)	Species = 4	7 (24)	Species = 4

Distribution of the isolates and OTUs in taxonomic groups and their abundance in the individual samples are displayed.

a: Operational Taxonomic Units b: Values in parenthesis corresponds to total number of microbial strains identified.

Total number of phylotypes observed:

Lab-reared adult male *A. stephensi *= 26

Lab-reared adult female *A. stephensi *= 18

### Analysis of the 16S rRNA gene clone library from lab-reared adult *A. stephensi*

One hundred clones were screened from each lab-reared adult male and female *A. stephensi *16S rRNA gene library, out of which 50 clones from each were analyzed further on the basis of sequencing results. The 16S rRNA gene sequencing data of isolates and clones were used to divide them into broad taxonomic groupings. The relative abundance or percent distribution of the taxonomic groups obtained in lab-reared adult *A. stephensi *is shown in Figure 1. Analysis of the 16S rRNA gene sequence revealed that the libraries were dominated by sequences related to the genus *Pseudomonas *and *Serratia *(71% of the clones examined). The majority of the cultured isolates and the 16S rRNA gene library clones belonged to the gammaproteobacteria class. Diversity of bacteria within the 16S rRNA gene libraries from lab-reared male and female *A. stephensi *was rather low, with relatively few phylotypes. Low bacterial diversity in *Anopheles *species by 16S rRNA gene sequencing has been reported, with six, two, and one bacterial species in *A. arabiensis*, *A. gambiae *sensu stricto, and *A. funestus*, respectively [16]. We detected few operational taxonomic units (OTU) within the gammaproteobacteria that were detected in other studies by 16S rRNA gene sequencing and bacterial isolation [10,16]. This difference may be due to the differences in microbial ecology which widens the view of the actual diversity residing in a system.

A total of 12 genera were identified, 7 from the lab-reared adult male and 5 from adult female *A. stephensi *16S rRNA library and used to assign each of the clones to taxonomic groups (Table 1). Cloning revealed that almost 50% of the sequences obtained in both the libraries were related to known bacteria, which fall within defined groups (bacteria/species). It can be seen that there are not much of the differences between isolates and the 16S rRNA gene library from lab- reared adult *A. stephensi *in the relative abundance of the different taxonomic groups. These appeared to reflect that except few isolates, microbial flora present in adult mosquitoes was more or less similar.

### Bacterial Community Structure

We grouped 16S rRNA gene sequences with its nearest neighbors (clone clusters) as shown by BLASTn search and clone clusters are comprised of one or more phylotypes. Sequences with more than 97% similarity were considered to be of the same OTUs. The frequencies of the OTUs obtained are shown in Table 1. A total of 22 phylotypes were observed, 15 from lab-reared male and 7 from female *A. stephensi *16S rRNA library. Whereas, by culturable methods 22 phylotypes were detected, 11 each from lab-reared male and female *A. stephensi*.

The most abundant phylotypes (71% in male, 37% in female) in the lab-reared adult *A. stephensi *16S rRNA libraries were closest matches to gammaproteobacteria (*Pseudomonas mendocina*, *Pseudomonas tolaasii*, *S. marcescens *and *Klebsiella *sp.) and CFB (*Elizabethkingia meningoseptica*, *C. meninqosepticum*, 37% in male and 29% in female mosquitoes). Almost same pattern is observed among culturable isolates, with gammaproteobacteria and CFB as major phylotypes detected. *Elizabethkingia meningoseptica *clones were observed (less frequently) only in adult 16S rRNA gene libraries, no culturable isolate was identified, whereas *C. meninqosepticum*, was detected in culturable as well as 16S rRNA gene clones among adult mosquitoes.

Second major phylotypes in lab-reared male 16S rRNA gene library belonged to alphaproteobacteria – *Agrobacterium tumefaciens *(13%) followed by unidentified class of bacteria (13%), none of the alphaproteobacteria and unidentified bacterium clones were detected from female 16S rRNA library. The degree of similarity of clone sequences and the 16S rRNA gene sequence of its closest relative in the database was in the range of 90–99%. The phylotypes indicated by culture-independent methods exhibited greater divergence and diversity than phylotypes recovered by culturing (Figure 1).

### Diversity of Cultured Bacteria from field-collected adult *A. stephensi*

#### Male *Anopheles stephensi*

**Figure 2:**
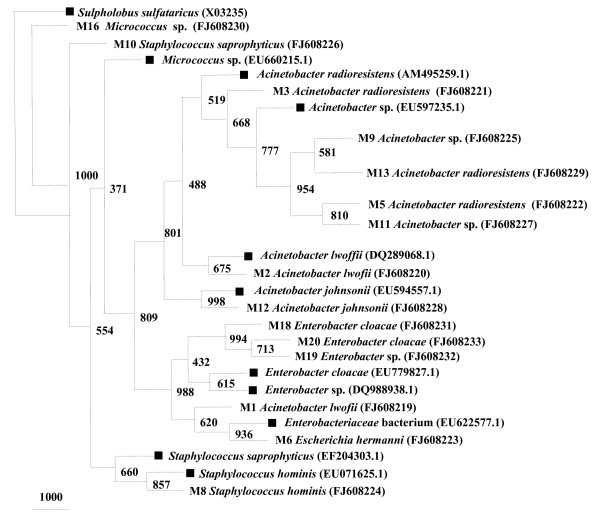
**Phylogenetic tree constructed for partial 16S rRNA gene of isolates cultured from field-collected male *A. stephensi***. Bootstrap values are given at nodes. Entries with black square represent generic names and accession numbers (in parentheses) from public databases. Entries from this work are represented as: strain number, generic name and accession number (in parentheses).

Analysis with the 16S rRNA gene sequence identified 17 different bacterial isolates by culture- dependent methods. The phylogenetic tree based on 16S rRNA gene placed the 17 different bacterial isolates, with their closest matches into 3 major bacterial phyla. The 16S rRNA gene sequences from a variety of phylogenetic groups are shown in Figure 2. In field-collected male *A. stephensi *3 major groups were, high G+C Gram-positive Actinobacteria, Gram-positive Firmicutes and gammaproteobacteria. Distinctive representative genera were; *Micrococcus *sp., *Staphylococcus hominis*, *S. saprophyticus*, *Acinetobacter *sp., *A. lwofii*, *A. radioresistens*, *A. johnsonii*, *Enterobacter *sp., *E. cloacae *and *Escherichia hermani *details of which are shown in Table 2. Sequences with more than 97% similarity were considered to be of the same OTUs. A total of 14 distinct phylotypes were identified from male *A. stephensi*. The frequencies of the OTUs obtained are shown in Table 2.

**Table 2:** Abundance of isolates and clones within the bacterial domain derived from the 16S rRNA gene sequences of isolates from field- collected *A. stephensi*.

Group	Adult Male Culturable		Adult Male Unculturable		Adult Female Culturable		Adult Female Unculturable		Larvae Culturable		Larvae Unculturable	
	
	OTU^a^	Matches	OTU	Matches	OTU	Matches	OTU	Matches	OUT	Matches	OTU	Matches
Cyano	-	-	-	-		-		-	-	-	1(1)	*Calothrix *sp.

Actino	1(1)^b^	*Micrococcus* sp.	-	-	-	-	-	-	-	-	1(1)	*Brevibacterium paucivorans*

CFB group	-	-	1(1)	*Flexibacteriaceae*	1(1)	*Chryseobacterium indologenes*	-	-	2(2)	*C. indologenes*	1(1)	*Dysqonomonas* sp.

Firmicutes	1(1)	*Staphylococcus hominis*	1(1)	*Bacillus *sp.	-	-	1(1)	*Leuconostoc citreum*	1(1)	*Bacillus *sp.	2(2)	*Staphylococcus* *cohnii*
	
	1(1)	*S. saprophyticus*	6(21)	*Paenibacillus alginolyticus*	-	-	-	-	1(1)	*B. cereus*	1(1)	*S. suis*
	
	-	-	1(1)	*P. chondroitinus*	-	-	-	-	1(1)	*B. firmus*	3(5)	*B. thermo* *amylovorans*
	
	-	-	7(31)	*Paenibacillaceae*	-	-	-	-	3(3)	*Exiguo* *bacterium*	1(1)	*Lactobacillus*

Beta-Proteo bacteria	-	-	1(1)	*Herbaspirillum sp.*	-	-	1(1)	*Achromobacter xylosoxidans*	-	-	3(5)	*Azoarcus *sp.
	
	-	-	-	-	-	-	-	-	-	-	1(1)	*Leptothrix *sp.
	
	-	*-*	-	-		-		-	-	-	1(1)	*Hydroxenophaga*
Gamma-Proteo bacteria	2(2)	*Acinetobacter*	1(1)	*Photorhabdus luminescens*	1(2)	*Acinetobacter*	2(4)	*Acinetobacter*	5(6)	*A. venetianus*	1(1)	*Enterobacter aerogenes*
	
	1(2)	*A. lwofii*	-	-	1(1)	*A. hemolyticus*	2(3)	*A. hemolyticus*	1(1)	*Aeromonas* *sobria*	1(1)	*Ignatzschineria *larvae sp.
	
	3(3)	*A. radioresistens*	-	-	3(4)	*A. radioresistens*	1(1)	*Acinetobacter* sp.	1(1)	*A. popoffii*	1(1)	*Enterobacter* sp.
	
	1(2)	*A. johnsonii*	-	-	1(1)	*Citrobacter freundii*	2(2)	*Pseudomonas putida*	4(4)	*P. anquilliseptica*	2(6)	*Serratia* sp.
	
	1(1)	*Enterobacter*	-	-	4(6)	*Enterobacter*	2(2)	*P. synxantha*	1(1)	*Pseudo* *xanthomonas*	1(1)	*Serratia* sp.
	
	1(2)	*E. cloacae*	-	-	14(15)	*E. cloacae*	1(1)	*Pseudomonas* sp.	4(4)	*Thorsellia anopheles*	2(3)	*T. anopheles*
	
	-	*-*	-	-	2(2)	*E. sakazaki*	8(23)	*S. marcescens*	2(2)	*Vibrio chlorae*	6(24)	*S. marcescens*
	
	2(2)	*Escherichia hermani*	-	-	1(1)	*E. hermani*	6(15)	*S. nematodiphila*	-	-	4(6)	*S. nematodiphila*
	
	-	-	-	-	-	-	1(1)	*S. proteamaculans*	-	-	-	-
	
	-	-	-	-	-	-	1(1)	*Xenorhabdus nematodiphila*	-	-	-	-
	
	-	-	-	-	-	-	1(1)	*Leminorella grimontii*	-	-	-	-
	
	-	*-*	-	-	-	-	2(4)	Uncultured	-	*-*	-	-
	
	-	-	-	-	1(1)	*Entero* *bacteriaceae*	1(1)	*Entero* *bacteriaceae*	-	-	-	-

Deinococcus	-	-	-	-	-	-	-	-	1(1)	*Deinococcus xinjiangensis*	2(4)	*D. xinjiangensis*

Uncultured	-	-	9(28)	Uncultured	-	-	4(8)	Uncultured	2(2)	Uncultured	1(1)	Uncultured

No match	3	No match^c^	15	No match	2	No match	10	No match	7	No match	1	No match

Total	14 (17)	Species = 10	27 (85)	Species = 8	29 (34)	Species = 10	36 (69)	Species = 16	29 (30)	Species = 14	36 (66)	Species = 20

Distribution of the clones and OTUs in taxonomic groups and their abundance in the individual samples are displayed. **a: **Operational Taxonomic Units, **b: **Values in parenthesis corresponds to total number of microbial strains identified, **c: **No significant similarity found (Sequences not included for analysis).

**Total number of phylotypes observed: **Field-collected adult male *A. stephensi *= 41,

Field-collected adult female *A. stephensi *= 65, Field-collected larvae of *A. stephensi *= 65.

A large proportion of the isolates, 82% was identified as gammaproteobacteria, where dominant genera were *Acinetobacter*, *Enterobacter *and *Escherichia*. The group of firmicutes constituted 12% of the total clones and was moderately occupied by *Staphylococcus hominis *and *S. saprophyticus*. High G+C Gram positive actinobacteria (*Micrococcus *sp.) was represented by a single clone OTU observed among 6% of total male isolates. It was showing less than 85% homology to the closest database match.

#### Male *Anopheles stephensi *16S rRNA gene library

**Figure 3:**
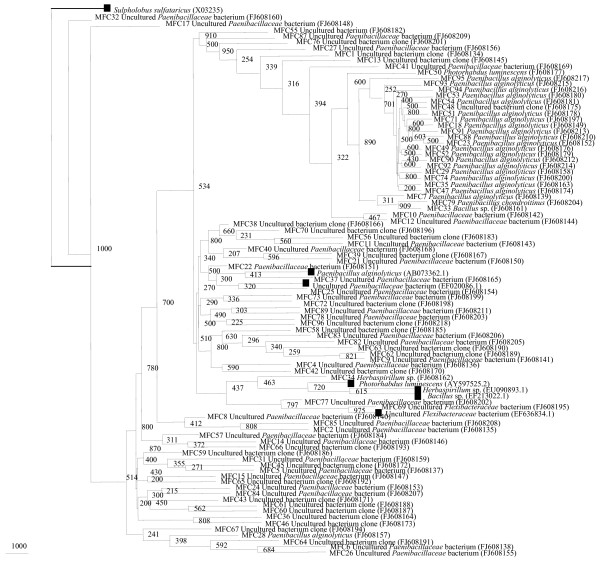
**Neighbor-Joining tree deduced from partial sequences of 16S rRNA gene clones from field-collected male *A. stephensi***. Bootstrap confidence values obtained with 1000 resamplings are given at the branch point. Entries with black square represent generic names and accession numbers (in parentheses) from public databases. Entries from this work are represented as: clone number, generic name and accession number (in parentheses).

A total of 150 clones were analyzed initially from 16S rRNA gene library of midgut content of field-collected male *A. stephensi*. The 16S rRNA gene sequencing placed the clones with their closest matches into 4 major bacterial groups: CFB, Gram-positive firmicutes, betaproteobacteria and gammaproteobacteria. In male *A. stephensi *16S rRNA gene library, Gram-positive bacteria, especially bacteria of the phylum *Firmicutes *dominated the flora. This is not in accordance with culture-based studies made in male *A. stephensi*. A total of 27 distinct phylotypes were identified from male 16S rRNA library clones (Table 2). The most frequently encountered sequences in this work originated from species of the genera: *Bacillus *sp., *Paenibacillus alginolyticus*, *P. chondroitinus*, and *Herbaspirillum *sp. These phylotypes were specific to the field-collected male midgut flora, as none of the species were identified in rest of the samples. *Bacillus *sp., *P. chondroitinus*, *Herbaspirillum *sp., and *Photorhabdus luminescens *were identified as single unique phylotypes (Table 2, Figure 3). The Good's coverage calculated for the 85 clones was 68.23% (Table 3).

**Table 3:** Comparison of the phylotype richness, diversity and evenness values of the isolates and 16S rRNA clones from lab-reared and field-collected *A. stephensi *mosquitoes.

Index	Lab-reared *A. stephensi*				Field-collected *A. stephensi*					
	
	Culturable		Unculturable		Culturable			Unculturable		
	
	M	F	M	F	M	F	L	M	F	L
**No. of isolates/clones**	18	16	24	24	17	34	30	85	69	66

**S^a^**	11	11	15	7	14	29	29	27	36	36

**H^b^**	1.74	1.84	2.14	1.97	2.75	2.93	3.21	2.93	3.15	3.49

**E^c^**	0.89	0.94	0.89	0.70	0.99	0.93	0.98	0.98	0.98	0.99

**C_ACE**	45	43	43	31	50	173	157	72	160	123

**C_Chao**	25	30	30	15	35	104	129	71	117	94

**C_Simpson**	0.013	0.011	0.08	0.54	0.017	0.02	0.02	0.11	0.11	0.06

**Good's Coverage**	39	32	38	71	18	15	13	69	49	46

The table lists the number of phylotypes, observed and estimated species richness, coverage and diversity indices for the culturables and 16S rRNA clone libraries from lab-reared and field- collected adult and larval *Anopheles stephensi *mosquitoes. Numbers were calculated with DOTUR program, OTUs were defined using a distance level of 3%.

The Shannon-Weiner diversity index [16] is calculated as follows:

a: S = (Phylotype richness): Total number of species in the sample.

b: H = Σ (pi) (log_2 _p - i), where p represents the proportion of a distinct phylotype relative to the sum of all phylotypes.

c: E = (Evenness) was calculated as follows: E = H/Hmax where Hmax = log_2 _(S)

C_ACE = ACE Coverage, C_Chao = Chao Coverage, C_Simpson = Simpson Coverage

Good's Coverage = [1 - (*n*/N)] × 100

Where *n *is the number of molecular species represented by one clone (single-clone OTUs) and N is the total number of sequences [54].

M: Adult Male *Anopheles stephensi*

F: Adult Female *Anopheles stephensi*

L: *Anopheles stephensi *Larvae

In all, 64% of the clones were found to belong to firmicutes, followed by 28% from unclassified class of bacteria (mainly uncultured *Flexibacteriaceae *and uncultured *Paenibacillaceae*) were also identified. CFB, betaproteobacteria and gammaproteobacteria, each constituted 1% of the total clones (Figure 1). It can be observed here that among culturable isolates gammaproteobacteria are the dominant group, whereas 16S rRNA gene clones were dominated by firmicutes. Both the approaches ("culture-dependent and culture-independent") have led to the identification of more number of genera in each sample as compared to single sample analysis.

#### Female *Anopheles stephensi*

**Figure 4:**
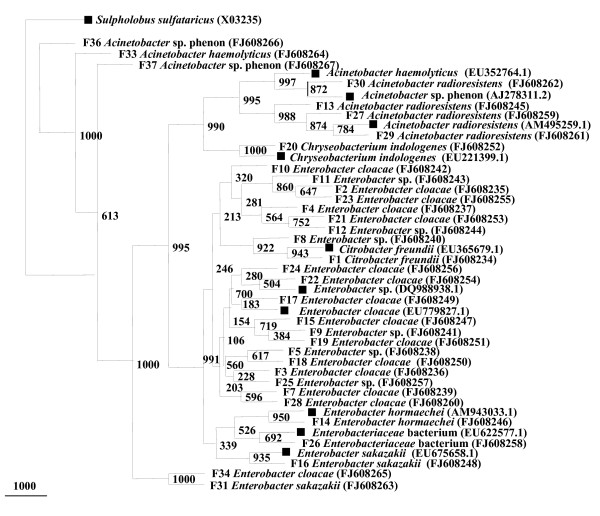
**Phylogenetic tree constructed for partial 16S rRNA gene of isolates cultured from field-collected female *A. stephensi***. Bootstrap values are given at nodes. Entries with black square represent generic names and accession numbers (in parentheses) from public databases. Entries from this work are represented as: strain number, generic name and accession number (in parentheses).

A total of 34 distinct isolates were identified from field-collected female *A. stephensi *midgut microflora. On the basis of phylogenetic tree 16S rRNA gene sequences were found to belong to major two bacterial phyla, gammaproteobacteria and CFB (Figure 4). The majority of the cultured isolates from field-collected and lab-reared adults belonged to the gammaproteobacteria class. A total of 29 bacterial OTUs were detected among female *A. stephensi *on the basis of 97% sequence similarity as a cut off value (Table 2). Sequences with more than 97% similarity were considered to be of the same OTUs. Representative genera of gammaproteobacteria were, *Acinetobacter *sp., *A. hemolyticus*, *A. radioresistens*, *Citrobacter freundii*, *Enterobacter *sp., *E. cloacae*, *E. sakazaki*, *Escherichia hermani *and *Enterobacteriaceae *bacterium. They constituted the largest proportion of 97%, among the total diversity. Out of the 29 distinct phylotypes observed, 28 were found to belong to class gammaproteobacteria only. Only single phylotype *Chryseobacterium indologenes*, from CFB was detected with 3% proportion from the total observed OTUs. None of the member from high G+C Gram-positive actinobacteria and Gram-positive firmicutes were observed, as in field-collected male *A. stephensi*. Similarly, none of the CFB group phylotypes were detected in female *A. stephensi*. Isolates belonging to genus *Acinetobacter *sp., *A. radioresistens*, *Enterobacter *sp., *E. cloacae *and *Escherichia hermani *were commonly observed in both male as well as female field-collected *A. stephensi*. These results are quite different from the data what we have observed in lab-reared adult *A. stephensi *(Figure 1).

#### Female *Anopheles stephensi *16S rRNA gene library

**Figure 5:**
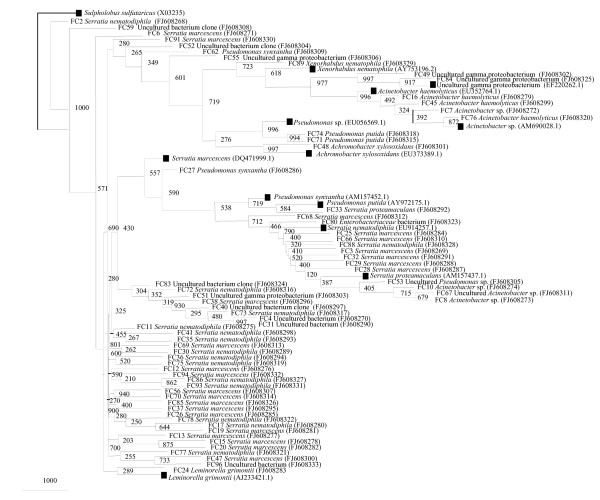
**Neighbor-Joining tree deduced from partial sequences of 16S rRNA gene clones from field-collected female *A. stephensi***. Bootstrap confidence values obtained with 1000 resamplings are given at the branch point. Entries with black square represent generic names and accession numbers (in parentheses) from public databases. Entries from this work are represented as: clone number, generic name and accession number (in parentheses).

A total of 100 clones were found positive for the insert and were partially sequenced. Of these, three were shown to be chimeras and were therefore not included for further analysis. The phylogenetic analysis of the remaining clones was done using partial 16S rRNA gene aligned homologous nucleotide sequences (Figure 5). The percentage distribution of the clones from the 16S rRNA gene library representing the microbiota of female *A. stephensi *midgut was determined (Table 2, Figure 1) On the basis of sequence similarity to the existing GenBank database entries, the clones were clustered together to form four major groups: Gram positive firmicutes, betaproteobacteria and gammaproteobacteria and the unidentified and uncultured bacteria group. The last group included all the uncharacterized and as yet uncultured bacteria. Thirty six distinct phylotypes were observed from female *A. stephensi *midgut 16S rRNA gene library.

In accordance with culturable isolates, 16S rRNA libraries were also dominated by gammaproteobacteria, constituting 86% of the total clones analyzed. Representative genera were: *Acinetobacter *sp., *A. hemolyticus*, uncultured *Acinetobacter *sp., *Pseudomonas putida*, *P. synxantha*, uncultured *Pseudomonas *sp., *Serratia marcescens*, *S. nematodiphila*, *S. proteamaculans*, *Xenorhabdus nematodiphila*, *Leminorella grimontii*, uncultured gamma proteobacteria and *Enterobacteriaceae *bacterium.

Unclassified group represented 12% of the total clones (90–98% similarity to closest database matches) whereas Gram-positive firmicute (*Leuconostoc citreum*) and betaproteobacteria (*Achromobacter xylosoxidans*) contributed 1% each to the total number of clones analyzed. *Leuconostoc citreum *is one of the most prevalent lactic acid bacteria, in a best-known Korean traditional dish. It can suppress the growth of pathogenic microorganisms such as *B. cereus*, *Listeria monocytogenes*, *Micrococcus luteus*, *P. aeruginosa *and *Salmonella enterica *serovar *typhimurium*. Its complete genome sequence may provide us with scientific insights into the probiotic effects of *L. citreum *and may lead to new biotechnological applications along with its significance inside mosquito midgut.

It is interesting to observe here that many of the single clone OTUs such as *Leuconostoc citreum*, *Achromobacter xylosoxidans*, *Pseudomonas synxantha*, *S. nematodiphila*, *S. proteamaculans*, *Xenorhabdus nematodiphila *and *Leminorella grimontii *were particularly present in female *A. stephensi *midgut microbial flora and was not present in either male or larval midgut microbial diversity.

#### *Anopheles stephensi *Larvae

**Figure 6:**
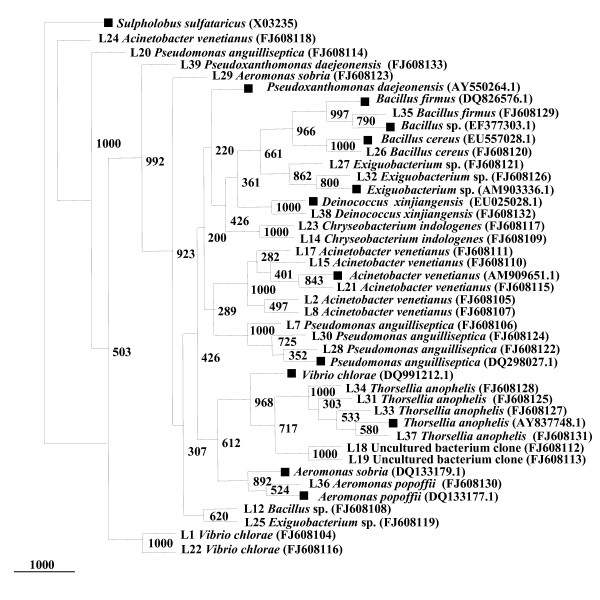
**Phylogenetic tree constructed for partial 16S rRNA gene of isolates cultured from field- collected *A. stephensi *larvae**. Bootstrap values are given at nodes. Entries with black square represent generic names and accession numbers (in parentheses) from public databases. Entries from this work are represented as: strain number, generic name and accession number (in parentheses).

Five major phyla, CFB, Gram-positive firmicutes, gammaproteobacteria, *Deinococcus-thermus *and unidentified class of bacteria were identified from 30 isolates of field-collected *A. stephensi *Larvae. A total of 29 phylotypes were observed with 97% similarity values as cut off. The 16S rRNA gene sequences from a variety of phylogenetic groups are shown in Figure 6. The majority of the cultured isolates (63%) from field-collected *A. stephensi *larvae were found to belonging gammaproteobacteria class. Distinct genera were *Acinetobacter venetianus*, *Aeromonas sobria*, *A. popoffii*, *Pseudomonas anquilliseptica*, uncultured *pseudoxanthomonas*, *Thorsellia anopheles *and *Vibrio chlorae*. Gram-positive firmicutes represented second abundant phylotypes (20% of the isolates) containing *Bacillus *sp., *B. cereus*, *B. firmus *and *Exiguobacterium *sp. CFB group (*Chryseobacterium indologenes*) and uncultured class of bacteria constituted an equal proportion of 7%. The degree of similarity of isolates and the 16S rRNA gene sequence of its closest relative in the database was in the range of 85–99%. Uncultured class of bacterial sequences obtained was related to unknown, possibly novel bacteria, which did not fall within defined groups (new bacteria/species). A single OTU was observed from *Deinococcus xinjiangensis *(Table 2).

It can be observed here that the majority of the cultured isolates from field-collected adults and larvae belonged to the gammaproteobacteria class with *Acinetobacter *as a common and dominant genus. Most of the sequence types were specific to larval samples only, such as *Aeromonas sobria*, *A. popoffii*, *Pseudomonas anquilliseptica*, uncultured *Pseudoxanthomonas*, *Thorsellia anopheles *and *Vibrio chlorae*. *Bacillus firmus*, *Exiguobacterium *sp. and *Deinococcus xinjiangensis *were not detected in either male or female midgut bacterial flora.

### 16S rRNA gene library analysis from *Anopheles stephensi *larvae

**Figure 7:**
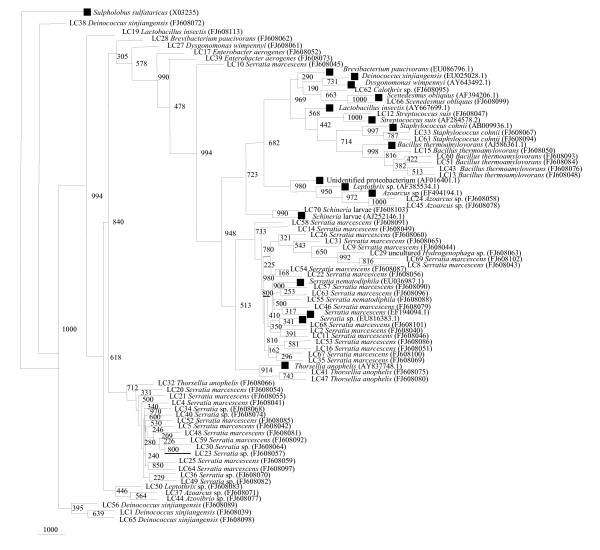
**Neighbor-Joining tree deduced from partial sequences of 16S rRNA gene clones from field-collected *A. stephensi *larvae**. Bootstrap confidence values obtained with 1000 resamplings are given at the branch point. Entries with black square represents generic names and accession numbers (in parentheses) from public databases. Entries from this work are represented as: clone number, generic name and accession number (in parentheses).

More than 100 clones were found positive for the insert and were partially sequenced, 80 of which were found to contain the amplified 16S rRNA gene. Of these, four sequences were shown to be chimeras, which were therefore not included for further analysis. The percentage distribution of the clones from the 16S rRNA gene library representing the microbiota of the midgut of *A. stephensi *larvae was determined (Table 2, Figure 7). The phylogenetic tree based on 16S rRNA gene placed the 16S rRNA gene library clones from field-collected *A. stephensi *larvae sample into 8 major groups, belonging to 19 different genera (Table 2). These groups were: Cyanobacteria, Actinobacteria, CFB group bacteria, Gram-positive Firmicutes, betaproteobacteria, gammaproteobacteria, *Deinococcus xinjiangensis*, and the unidentified and uncultured bacteria group. Larval midgut microbial flora was the found to be most diverse as compared to adult mosquito midgut diversity. Cloning revealed that almost 50% of the sequences obtained in library were not related to the known bacteria. Since the percent similarity with the reported closest database matches are less than 97%, these may be categorized among the new bacteria/species. A total of 36 phylotypes were observed from 16S rRNA library based on their less than 97% similarity.

The most abundant phylotypes were closest matches to gammaproteobacteria, constituting 65% of the clones. Distinct genera were *Enterobacter aerogenes, Ignatzschineria *larvae sp., uncultured *Enterobacter *sp., *Serratia *sp., uncultured *Serratia *sp., *S. marcescens*, *S. nematodiphila *and *Thorsellia anopheles*. Gram-positive firmicutes contributed 14% of distinct phylotypes from groups of *Staphylococcus cohnii*, *Streptococcus suis*, uncultured *B. thermoamylovorans *and uncultured *Lactobacillus *sp. The inability to detect *Bacillus *sp. in clone libraries despite their presence on plates was observed among larvae samples. 11% of the clones were found to belong to betaproteobacteria, mainly *Azoarcus *sp., *Leptothrix *sp. and uncultured *Hydroxenophaga *sp. *Deinococcus xinjiangensis *was identified as single clone OTUs among 6% of the clones. Cyanobacteria, Actinobacteria, CFB group and uncultured class of clones represented 1% of the single clone OTUs as *Calothrix *sp., *Brevibacterium paucivorans*, uncultured *Dysqonomona *sp. and uncultured bacterium (Figure 1). The degree of similarity of clone sequences and the 16S rRNA gene sequence of its closest match in the database were in the range of 85–98%. It was very interesting to observe that the individual libraries harbored many sequence types unique to that library and sample, so the even single data set provides a better estimate of the total diversity in all the samples. Among the lab-reared and field-caught mosquito midgut bacteria *Chryseobacterium*, *Pseudomonas *and *Serratia *sp. were found to be overlapping in adult female and larval mosquitoes, whereas no genera were found to be overlapping in adult male *A. stephensi*.

#### Uncultured groups and "Novel" lineages

Results of Jukes-Cantor evolutionary distance matrix suggested that the vast majority of the sequences were different strains of known and unknown species and may represent new species within the genus of different phylum. Many 16S rRNA gene sequences from field-collected male *A. stephensi *(M1, M6, M10, M16) (Figure 2) and many clusters of different phylotypes in female *A. stephensi*, such as F31, F33, F34, F36, F37 (Figure 4) were very distinct from those of cultured organisms present in the NCBI database. Larval *A. stephensi *sequences (L12, L15, L18, L19, L20, L24, L29 and L39, Figure. 6) were also found to be deep branching in tree with low bootstrap values, which suggests a high genetic diversity. These did not appear to fall within defined groups and subgroups and may represent "novel" species. Many of such novel isolates have been reported earlier by 16S rRNA gene-based identification of midgut bacteria from field-caught *A. gambiae *and *A. funestus m*osquitoes which have revealed new species related to known insect symbionts [16]. Further characterizations of these isolates are in progress. Few of them could be identified only to the family level (*Enterobacteriaceae*, *Paenibacillaceae *and *Flexibacteriaceae*) (Table 2). The family *Enterobacteriaceae *contains various species previously described as insect symbionts in mosquito midgut screens [9,10,28-30]. From this study it is proposed that environmental conditions (for example, laboratory and field) provide a specific ecological niche for prolonging survival of diverse and "novel" microbial species.

#### Diversity Index Analysis

Diversity index quantifies diversity in a community and describe its numerical structure. The analysis indicated that most of the bacterial diversity has been sufficiently covered (Table 3). Shannon Weaver diversity index (H) for culturable isolates of lab-reared male and female *A. stephensi *were 1.74 and 1.84 and for uncultivable clones was calculated to be 2.14 and 1.97 respectively. Species evenness (E) for the culturables from lab-reared male and female *A. stephensi *were 0.89 and 0.94 and for unculturable flora was 0.89 and 0.70 respectively.

These index values varied significantly in field-collected male and female *A. stephensi*. Shannon's diversity index (H) for culturable diversity of field-collected male and female *A. stephensi *was 2.75 and 2.93 and for uncultivable diversity was calculated to be 2.93 and 3.15 respectively. Species evenness (E) for the culturable isolates from field-collected male and female *A. stephensi *were 0.89 and 0.94 and for unculturable diversity were 0.89 and 0.70 respectively.

Shannon's index (H) and species evenness values were observed to be comparatively higher for field-collected *A. stephensi *larvae (3.21 for culturable subset and 3.49 for 16S rRNA library clones). Species evenness (E) for the culturable isolates from field-collected *A. stephensi *larvae was 0.98 and for unculturable diversity was estimated to be 0.99. In a recent study on bacterial diversity in the midgut of field-collected adult *A. gambiae *as measured by the Shannon- Weaver diversity index, (H) ranged from 2.48 to 2.72, which was slightly higher than those observed for bulk water (1.32–2.42). Bacterial diversity indices in all midgut samples were within the range of H values observed for water (larvae, H = 2.26–2.63; adults, H = 2.16–2.52) [13]. These values indicate that the diversity and evenness are quite higher in our samples. The evenness and dominance values approximate to the maximum possible values, as most of the sequence types were recovered only once. The sample coverage using Good's method for the male, female and larvae (individual 16S rRNA gene libraries) ranged from 38 to 71%.

Thus, Shannon and Simpson diversity indices suggested higher diversity in the field- collected adult male, female and larval midgut flora than the lab-reared adult male and female *A. stephensi*. The Shannon index gives more weight to the rare species and Simpson to the dominant [31], but in this case they were quite concordant. The ACE and Chao estimators did not agree with Shannon and Simpson in all cases. The Chao estimator takes into account only singletons and doubletons, ACE uses OTUs having one to ten clones each [31,32]. The ACE and especially Chao are dependent of the amount of singletons and the discrepancies with the diversity indices are most probably due to different amounts of singletons in the libraries. Higher coverage's have been reported with libraries from human sources, (as high as 99%) which may be due to the larger number of sequenced clones in these studies [33,34].

**Figure 8:**
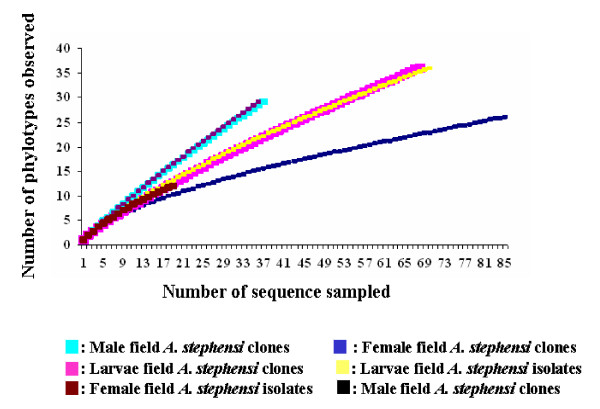
**Rarefaction curve from DOTUR analysis using partial 16S rRNA gene sequences of isolates and clones from field-collected *A. stephensi *(male/female/larvae) mosquitoes**. 16S rRNA gene sequences were grouped in to same OTUs by using 97% similarity as a cut off value.

In lab-reared and field-collected adult and larval midgut flora of *A. stephensi *investigated in this work, the estimated OTU number was 215 using 97% sequence identity as the criterion in DOTUR, using the pooled sequence data from all isolates and clones. The ACE estimate for the individual libraries varied from 50 to 173 (Table 3). The individual libraries harbored many sequence types unique to that library, such that, even pooled data set provides a better estimate of the total diversity. Rarefaction curve analyses (Figure 8) revealed that field-collected *A. stephensi *male, female and larvae midgut microbial flora ("cultured and uncultured microbes") consist of a vast diversity. In clone libraries, with increasing numbers of sequences, the number of OTUs increases, until saturation is reached. In order to cover total diversity a large number of sequences need to be sampled. However, the present analysis indicates that it is more or less sufficient to give an overview of dominating microbial communities for these two, lab-reared and field- collected environments.

## Discussion

We have identified the richness and diversity of microbes associated with lab-reared and field- collected mosquito, *A. stephensi*. Malaria transmitting vector *A. stephensi *occupies several ecological niches and is very successful in transmitting the parasite. Characterization of gut micobes by "culture-dependent and culture-independent" methods led to the identification of 115 culturable isolates and 271 distinct clones (16S rRNA gene library). The dominant bacteria in field-captured *A. stephensi *adult male were uncultured *Paenibacillaceae *family bacteria, while in larvae and female mosquitoes the dominant bacteria was *Serratia marcescens*. In lab-reared adult male and female *A. stephensi *bacteria, *Serratia marcescens *(61 to 71% of isolates/clones) and *Cryseobacterium meninqosepticum *(29 to 33% of isolates/clones) were found to be abundant.

Almost 50% isolates and 16S rRNA gene clones identified from field-collected adult and larvae *A. stephensi*, displayed 16S rRNA gene similarity to unidentified bacterium clones in public databases (NCBI, RDP-II). 16S rRNA gene sequences of majority of these isolates and clones displayed sequence similarities to cultured or the uncultured bacteria of gammaproteobacteria group. Recovery of many isolates and 16S rRNA clones belonging to the genus *Acinetobacter*, from field-collected adult male, female and larvae of *A. stephensi *indicate that gammaproteobacteria may form a significant proportion of the *A. stephensi *midgut microbiota. The presence of *Exiguobacterium *sp. bacterium related to activated sludge treatment probably reflects the ecological niche of larvae and the metabolic diversity of gammaproteobacteria and other bacterial groups [35-38]. A careful comparative analysis of breadth of diversity of microbes reported from other mosquito species reveals preponderance of bacteria, *Aeromonas, Acinetobacter*, *Enterobacter *and *Pseudomonas *in adult *A. stephensi *midgut flora. These bacterial species have also been identified from the midgut of other *Anopheles *sp., [28,39-41] suggesting that at least a fraction of mosquito midgut inhabitants could be common for different mosquito species inhabiting the similar environment and may represent evolutionary conservation of association of gut vector biology.

The transition from larvae to adult is a metabolically dynamic and complex process. It is likely that the gut-associated flora plays some role in facilitating this transition. The gut during larvae to adult transition is believed to undergo sterilization process and adults recruit new microbiota. Our results revealed that the gut sterilization is not complete during transition and certain bacteria are retained (*Acinetobacter*, *Bacillus*, *Enterobacter*, *Staphylococcus*, *Pseudomonas*, *Cryseobacterium *and *Serratia *sp). These bacterial species do not become dominant during adult maturation and remain in low abundance except *Cryseobacterium *and *Serratia *sp., which were relatively high in lab-reared adult male, female and field-collected larvae and adult female *A. stephensi*. *Acinetobacter *and *Enterobacter *sp. were retained by both male and female field-collected *A. stephensi*. It is interesting to observe here that *Bacillus *and *Staphylococcus *sp. were exclusively retained by adult field-collected male *A. stephensi*, whereas, *Cryseobacterium*, *Pseudomonas *and *Serratia *sp. were retained by adult field-collected female *A. stephensi*. Adult male and female mosquitoes are anisomorphic and have different feeding habits. The gut flora is known to help in various physiological processes including digestion. The difference in gut flora might help in digestion of different types of food in male and female mosquitoes. Female mosquitoes are anautogenous, i.e., they require blood meal for ovarian development, which also supplies loads of microbial flora while male mosquitoes never take blood. This may be the reason for the observed more diverse gut flora in adult female than in the male mosquitoes.

It is observed that the bacterial diversity in field-collected mosquitoes, whether male or female, was much more than that of lab-reared mosquitoes. Under laboratory conditions, the mosquitoes were reared in hygienic and controlled conditions whereas, reverse is true for the field conditions. Hence, the larvae in field are more exposed to the microbial flora of the open water than their counterparts in the laboratory. Larvae being filter feeders ingest the water in immediate vicinity irrespective of their preference. Similarly, adult mosquitoes feed on uncontrolled natural diet, while laboratory-reared mosquitoes were fed with sterile glucose solution and resins. Even the blood offered to female mosquitoes in laboratory is from infection-free rabbit; on the other hand, the blood meal in field is good source of various infections. Thus, field-collected mosquitoes have more chances of having diverse gut flora as was observed.

Mosquitoes are known to elicit specific immune responses against parasites [3,4,42]. Some of these immune responsive genes are expressed in response to bacteria and this raises the possibility that the presence of specific bacteria in the gut may have an effect on the efficacy at which a pathogen is transmitted by a vector mosquito [9]. In previous studies of lab-reared *A. stephensi *adults, it was demonstrated that great number of *S. marcescens *were found in the midgut of the insects, but was not found in larvae and pupae [10]. In another study, it was observed that *Plasmodium vivax *load in *A. albimanus *mosquitoes co-infected with *E. cloacae *and *S. marcensces *were lower (17 and 210 times respectively) than control aseptic *A. albimanus *mosquitoes with *Plasmodium vivax *infection (without *E. cloacae *and *S. marcensce*). In our study, we also observed that a relatively high number of *S. marcescens *(35 isolates from lab-reared male/female and 48 clones from field-collected female/larvae) were identified from lab and field- populations of *A. stephensi*. However, none *S. marcescens *species were identified from field- collected male *A. stephensi*. At this point it is premature to draw correlation between the occurrences of *S. marcensce *and pathogenecity or vector load. However, previous reports suggest that mortality in *S. marcensces*-infected *A. albimanus *mosquitoes was 13 times higher compared with the controls [12].

The present study assumes importance in the light of earlier studies which suggested that the composition of midgut microbiota has a significant effect on the survival of dengue (DEN) viruses in the gut lumen [43]. The overall susceptibility of *Aedes aegypti *mosquitoes to dengue viruses increased more than two-folds, with the incorporation of bacterium *Aeromonas culicicola*. However, the increase in susceptibility was not observed when the antibiotic-treated *A. aegypti *mosquitoes were used, indicating that *A. aegypti *mosquito midgut bacterial flora plays a role in determining their capacity to carry viral load to the virus [43]. It has also been proposed that *Wolbachia *strains might be used to skew *A. aegypti *mosquito population life span, thereby reducing pathogen transmission without eradicating mosquito populations [2]. Furthermore, studies involving the effect of midgut bacterial flora have indicated that the incorporation of the *Pseudomonas *and *Acinetobacter *isolates in the mosquito blood meal resulted in an increased vector load of parasite of *Culex quinquefasciatus *towards virus infections [44]. It has also been shown in lab-reared *Drosophila melanogaster *that genetic differences promote pathological gut bacterial assemblages, reducing host survival. There results imply that induced antimicrobial compounds function primarily to protect the insect against the bacteria that persist within their body, rather than to clear microbial infections and thus they directly benefit the insect survival [45]. Malaria-mosquito combination is believed to have been around for thousands of years. It is likely that acquired microflora permitted the maintenance of parasite in mosquito. The microbes could be benefiting mosquito by protecting against pathogenic bacteria or lowering the innate immunity of mosquito against parasite. It has been reported that reduction in the normal bacterial flora in the mosquito midgut increases *Plasmodium falciparum *infection rates in experimentally infected *Anopheles *mosquitoes [41]. Interactions between midgut bacteria and malaria parasites in wild mosquito populations could explain how the vector potential for malaria parasite transmission is modulated/influenced by environmental factors such as acquisition of different types of bacteria.

The results obtained from our study and from view of previous studies it is indicated that colonization of bacteria in mosquitoes occurs early during their development. It is reasonable to assume that infection of mosquitoes occurs by acquisition of different bacterial species from the environment. The midgut bacterial infection in mosquito field-populations may influence *P. vivax *transmission and could contribute to understanding variations in malaria incidence observed in different area. To the best of our knowledge, this is the first attempt of comparative cataloguing the midgut microbiota of a parasite transmitting vector *A. stephensi *from lab-reared and field- collected adult and larvae using "culture-dependent and independent methods". Most of the previous studies of midgut flora of *Anopheles *mosquitoes exclusively utilized culture-dependent methods for screening. By including culture-independent method, we obtained a broader picture of the mosquito midgut flora. These microbes represent a potential resource that could be employed in mechanisms to interfere with mosquito vector development and in interrupting parasite development.

## Conclusion

This work demonstrates that the microbial flora of larvae and adult *A*.*stephensi *midgut is complex and is dominated by gammaproteobacteria and Gram-positive firmicutes species. The dominant phylotypes most probably originated from midgut inhabitants. A sex specific variation was observed, this being reflected in the proportional changes of the microbial phyla, as well as at the species level. Identification methods detected a high microbial diversity among *A. stephensi *adult and larval midgut. The micro flora of the investigated *A. stephensi *adults and larvae differed statistically and differences between the larval microbial diversity was more pronounced than the differences noted between *A. stephensi *male and female culturable and unculturables. This work provided basic information about bacterial diversity in midgut of lab-reared and field-caught *A. stephensi *male female and larval species and its population dynamics and hence, qualitative information about the total bacterial exposure in midgut environment. Our future work will include characterization of the different sources of microbes and a quantitative assessment of the different microbial taxa. It is promising that several of the isolates are Gram-negative gammaproteobacteria, for which there are well established means of genetic modification. All of the bacterial isolates from this study will be further evaluated for their suitability as paratransgenic candidate.

## Methods

### Maintenance of *Anopheles stephensi*

Cyclic colonies of *Anopheles stephensi *were maintained in a mosquitarium maintained at 28 ± 2°C and 70–80% humidity. Adult mosquitoes were offered raisins and 1% glucose solution as a source of energy. Female mosquitoes were allowed to feed on caged rabbit for their ovarian development. Eggs were collected in filter paper lined plastic bowls half filled with de-ionized water and left undisturbed for two days to allow the eggs to hatch. Larvae were cultured in enamels trays and were fed upon mixture of dog biscuit and yeast extract in 3:1 ratio. Following pupation, the pupae were transferred to accordingly labeled cages for emergence of adults.

### Collection of mosquitoes and isolation of bacterial flora from midgut

IV instar anopheline larvae were collected thrice from cement tanks in District Jhajjar, Haryana, India (28°37'N and 76°39'E). The larvae were brought to the laboratory in Delhi within two hours of collection and those that are morphologically identified as *Anopheles stephensi *were pooled [46]. The larvae were surface sterilized for 5 sec. in 95% ethanol [28]. The larval guts were dissected aseptically in laminar hood using sterile entomological needles underneath a stereo microscope. The dissected midguts were transferred to the 100 μl of sterile phosphate-buffered solution (PBS) and were grounded to homogeneity.

For studying the microflora of adult mosquito midgut, the IV instar larvae were allowed to emerge in the adult mosquitoes and the females and males were separated based on their morphological differences. The midguts of both the sexes were aseptically dissected as described for the IV instar larvae. Similarly the lab-reared adult male and female *Anopheles stephensi *mosquitoes were also dissected to study the gut flora. Each midgut extract consisted of a mean number of 24, 25 and 30 pooled midguts of adult male, female and larvae respectively. Midgut extracts were stored in a -80°C deep freezer until further analysis.

### Isolation of Bacteria

#### Culture-Dependent Methods

Microbial strain isolation protocol followed addition of 1 ml of the each sample to 5 ml of trypticasein soy agar (TSA) and LB agar medium, (HiMedia, India) and incubated at 37°C, 200 rpm for 24 h–48 h. One hundred micro liters of these samples were spread on to TSA and LB agar plates (2% agar was added to the medium). A 100 μl aliquot from these samples was further serially diluted up to 10^-6 ^and plated onto TSA and LB agar. Incubations were done at 37°C for 24 h–48 h. This nutrient rich media supports growth of dominating and even supporting group population of microbes.

The initial number of 40 isolates was reduced to 20 colonies, selected randomly after a first round of screening based on colony characteristics (involving colony size, shape, color, margin, opacity, elevation, and consistency) and the morphology of isolates based on Gram's staining. The colonies on TSA and LB agar are expected to represent the heterotrophic bacterial population associated with both laboratory-reared and field-collected mosquitoes. This resulted in around 20–30 isolates from each sample. Single distinct colonies of isolates were picked and streaked on fresh TSA plates. Isolates were sub-cultured three times before using as pure culture.

#### Identification of bacterial isolates

Bacterial genomic DNA was isolated by colony PCR protocol. 16S rRNA gene was amplified using 16S universal primers as reported by Lane *et al*. (1991) PCR reactions were performed under the following conditions: Initial denaturation at 94°C for 1 min, followed by 30 cycles of 94°C for 1 min, annealing at 55°C for 1 min 30 sec, 72°C for 1 min and a final extension at 72°C for 10 min [47]. Partial 16S rRNA gene (600 to 900 bp product) was amplified using forward primer 27F 5'-AGAGTTTGATCCTGGCTCAG-3' and reverse primer 1492R 5'-TACGGCTACCTTGTTACGACTT-3'. The presence and yield of PCR product was determined on 1% agarose gel electrophoresis at 200 V for 30 min in 1× Tris-acetate-EDTA buffer and stained with ethidium bromide. The PCR products were purified using QIAquick gel extraction kit (Qiagen, Germany) and were partially sequenced using universal primers.

#### Screening of isolates on the basis of antibiotic-sensitivity assay

One hundred distinct isolated colonies from both lab-reared and field-collected mosquitoes were grown individually in LB medium at 37°C, 200 rpm for 24 h–48 h. One hundred micro liter bacterial culture (O.D_600_~1.0; 10^5 ^CFU) was spread on LB plates. Each isolate was tested against 12 different antibiotic discs of known concentrations: Ampicillin (Amp) 25 mcg, Carbenicillin (Car) 100 mcg, Chloramphenicol (Chl) 10 mcg, Gentamycin (Gen) 10 mcg, Kanamycin (Kan) 30 mcg, Nalidixic acid (Nal) 30 mcg, Penicillin G (Peni) 10 units, Polymyxin B (Poly) 100 units, Rifampicin (Rif) 15 mcg, Streptomycin (Str) 10 mcg, Tetracyclin (Tet) 10 mcg and Vancomycin (Van) 10 mcg were equidistantly placed on three NA plates at the rate of 4 discs per plate. Plates were incubated overnight at 37°C. Zone of inhibition of bacterial growth was measured (diameter in mm) and on the basis of zone of inhibition, isolates were segregated [38]. The strains were distinguishable at a preliminary level on the basis of response to all the 12 different antibiotics [see Additional file 1].

#### Determination of metabolic characteristics

Different isolates were patched individually onto selective media such as LB agar (as control), casein hydrolysate (1%), starch (1%), tributyrin (1%) and to identify their abilities to produce amylase, lipase and protease activity, respectively. All the plates were incubated at 37°C for 24–48 h. These activities were checked by observing for a zone of clearing around each bacterial isolate. For protease activity, plates containing casein hydrolysate were visualized by coomassie staining of the plates. For starch, the zone of clearing was observed after flooding the plates with iodine solution. Relative enzyme activity was calculated by finding the ratio of zone of clearing (mm) and size of the bacterial colony (mm).

## Culture-Independent Method

### 16S rRNA gene library construction

#### Total DNA isolation

Total microbial DNA was extracted by adapting minor modifications in the protocol described by Broderick *et al*. (2004) [48]. Midgut extracts were thawed and 600 μl of Tris-EDTA (TE) (10 mM Tris-HCl [pH 8.0], 1 mM EDTA) was added to each tube. The contents of the tube were then sonicated for 30 sec. as described earlier to separate bacterial cells from the gut wall and 537 μl of TE was removed and placed in a new 1.5 ml microcentrifuge tube. The sample was sonicated under the same conditions for 45 s to break open bacterial cells and was mixed thoroughly with 60 μl of 10% sodium dodecyl sulfate and 3 μl of 50 mg of proteinase K/ml and was incubated for 1:30 h at 37°C. Each tube was mixed with 100 μl of 5 M NaCl prior to the addition of 80 μl of 10% cetyltrimethyl ammonium bromide-5 M NaCl. The sample was mixed thoroughly and incubated at 65°C for 30 min. DNA was extracted with equal volumes of chloroform-isoamyl alcohol (CIA) (24:1 [vol/vol]) and phenol CIA (25:24:1 [vol/vol/vol]). DNA was precipitated with isopropanol and recovered by centrifugation. Pellets were resuspended in 100 μl of TE buffer. DNA concentration and purity was determined by absorbance ratio at 260/280 nm, and the DNA suspension was stored at -20°C until it was used for PCR and further analysis.

#### PCR amplification

Bacterial 16S rRNA gene from total DNA were amplified by PCR in a reaction mixture (50 μl) containing (as final concentration) 1× PCR buffer, with 2 mM MgCl_2_, 200 μM of each dNTPs, DNA (50 ng), 2 μM each of forward primer 27F 5'-AGAGTTTGATCATGGCTCAG-3' and reverse primer 1492R 5'-TACGGCTACCTTGTTACGACTT-3' [47] and 2.5 units of Taq DNA polymerase (Real Biotech Corporation, India). The reaction mixture was incubated at 94°C for 5 min for initial denaturation, followed by 30 cycles of 95°C for 30 sec, 53°C, 55°C or 58°C for 90 sec, 72°C for 2 min 30 sec and a final extension at 72°C for 10 minutes. All reactions were carried out in 0.2 ml tubes in an ABI Thermal Cycler. PCR product of the three annealing temperatures were pooled and was examined by electrophoresis on 1% agarose gels containing ethidium bromide. The amplified product was pooled and purified using gel band extraction kit (Qiagen, Germany).

#### Cloning of Bacterial 16S rRNA gene

16S rRNA gene clone libraries were constructed by ligating PCR product into pGEM-T easy vector system (Promega, USA) according to the manufacturer's instructions. The ligated product was transformed into *E*. *coli *DH5α. Transformants were grown on LB plates containing 100 μg mL^-1 ^each of ampicillin, X-gal and Isopropyl β-D-1-thiogalactopyranoside. Single white colonies that grew upon overnight incubation were patched on LB Amp plates. Plasmid DNA was isolated from transformants by plasmid prep kit (Axygen, USA). All clones in libraries of approximately 100 clones from each lab-reared and field-collected adults were sequenced.

#### DNA sequencing data analysis

Sequencing reactions were performed using the Big Dye reaction mix (Perkin-Elmer Corp.) at Macrogen Inc. South Korea. Purified plasmid DNA was initially sequenced by using the primers T7 and SP6, which flank the insert DNA in PGEM-T easy vector. DNA from cultured strains were sequenced by using 27F and 1492R primers. All partial 16S rRNA gene sequence assembly and analysis were carried out by using Lasergene package version 5.07 (DNASTAR, Inc., Madison, Wis. USA). Partial 16S rRNA gene sequences were initially analyzed using the BLASTn search facility. Chimeric artifacts were checked using CHECK_CHIMERA program of http://www.ncbi.nlm.nih.gov/blast/blast.cgi RDP II analysis software http://rdp.cme.msu.edu/[49,50] and by another chimera detection program "Bellerophon" available at http://foo.maths.uq.edu.au/~huber/bellerophon.pl[37,51,52]. The sequences were submitted to the NCBI (National Centre for Biotechnology and Information) and GenBank for obtaining accession numbers.

#### Phylogenetic tree construction

All the sequences were compared with 16S rRNA gene sequences available in the GenBank databases by BLASTn search. Multiple sequence alignments of partial 16S rRNA gene sequences were aligned using CLUSTAL W, version 1.8 [53]. Phylogenetic trees were constructed from evolutionary distances using the Neighbor-Joining method implemented through NEIGHBOR (DNADIST) from the PHYLIP version 3.61 packages [54]. The robustness of the phylogeny was tested by bootstrap analysis using 1000 iterations. *Sulpholobus solfataricus *(Accession number X03235) was selected as an out group [37]. Trees generated were analyzed with the TREEVIEW program [55]. Accession numbers of all isolates and clones can be viewed in respective phylogenetic tree. All of the sequences have been submitted to the NCBI (National Centre for Biotechnology and Information) GenBank sequence database. The accession numbers are the following; sequences from laboratory-reared adult male and female *A. stephensi *(female clones F1–F24): (FJ607957–FJ607980), (Female isolates 1F-16F): (FJ607981–FJ607996), (male isolates 1M-20M): (FJ607997–FJ608014), (male clones LMC1–LMC24): (FJ608015–FJ608038). Accession numbers from field caught adult male, female and larvae of *A. stephensi *are the following; (larvae clones LC1–LC70): (FJ608039–FJ608103), (larvae isolates L1–L39): (FJ608104–FJ608133), (male clones MFC1–MFC96: (FJ608134–FJ608218), (male isolates M1–M20): (FJ608219 – FJ608233), (female isolates F1–F37): (FJ608234–FJ608267), (female clones FC2–FC96): (FJ608268–FJ608333).

#### Richness Estimation by DOTUR

Distance-based operational taxonomic unit and richness (DOTUR) was used to calculate various diversity indices and richness estimators. Sequences are usually grouped as operational taxonomic units (OTUs) or phylotypes, both of which are defined by DNA sequence. A genetic distance is approximately equal to the converse of the identity percentage. DOTUR, assigns sequences accurately to OTUs or phylotypes based on sequence data by using values that are less than the cutoff level. 16S rRNA clone sequences were grouped into same OTUs by using 97% identity threshold. The source code is available at http://www.plantpath.wisc.edu/fac/joh/dotur.html[56]. A PHYLIP http://evolution.genetics.washington.edu/phylip.html[54] generated distance matrix is used as an input file, which assigns sequences to OTUs for every possible distance. DOTUR then calculates values that are used to construct rarefaction curves of observed OTUs, to ascertain the relative richness between culturable isolates and 16S rRNA gene libraries. In this study we used DOTURs dexterity by analyzing, culturable isolates and 16S rRNA gene libraries constructed from lab-reared and field-collected *A. stephensi*.

The Shannon-Weiner diversity index is [18,37] calculated as follows:

H = Σ (pi) (log_2 _p - i), where p represents the proportion of a distinct phylotype relative to the sum of all distinct phylotypes.

Evenness (E) was calculated as: E = H/Hmax where Hmax = log_2 _(S)

Richness (S): Total number of species in the samples, which are equal to the number of OTUs calculated above. The sample calculations are provided in the manual on the DOTUR website [56].

Coverage was calculated by Good's method, according to which the percentage of coverage was calculated with the formula [1 - (*n*/N)] × 100, where *n *is the number of molecular species represented by one clone (single-clone OTUs) and *N *is the total number of sequences [57].

#### Rarefaction curve for comparison of diversity

To compare the bacterial diversity of lab-reared and field-collected mosquito samples, a large number of clones were analyzed and a cutoff value of 97% was used for OTUs. To obtain a phylogenetic relationship between the various phylotypes, one representative member of each phylotype was selected. To determine if the number of clones analyzed in lab-reared and field- adapted adults were representative for the each bacterial community, a table was made in which each OTU was listed as many times as its observed frequency. Rarefaction curve was generated by plotting the number of OTUs observed against number of sequences sampled [55].

## Authors' contributions

AR performed the microbial culture, metagenome DNA isolation, 16S library construction, molecular phylogenetic analyses, statistical data interpretation and wrote the manuscript. AS collected mosquitoes from the field and identified *A. stephensi*, was involved in rearing of mosquitoes in mosquitarium, tissue dissection and processing of samples. RR contributed in design of the study and sampling. TA maintained *A. stephensi *mosquitoes in laboratory and was involved in tissue dissection and sample processing. RKB designed and supervised the study, edited the manuscript. All authors read and approved the final manuscript.

## Supplementary Material

Additional file 1 **Antibiotic sensitivity assay of microbial strains isolated from *A. stephensi *midgut**. The data provided represents the antibiotic response of strains isolated from *A. stephensi *midgut against selected class of antibiotics. Click here for file
