# Supplementary material for: Bacterial diversity analysis of larvae and adult midgut microflora using culture-dependent and culture-independent methods in lab-reared and field-collected Anopheles stephensi-an Asian malarial vector
Source: BMC Microbiol. 2009 May 19;9:96. doi: 10.1186/1471-2180-9-96 (PMC2698833; doi:10.1186/1471-2180-9-96)
Supplement: Additional file 1 — Antibiotic sensitivity assay of microbial strains isolated from A. stephensi midgut. The data provided represents the antibiotic response of strains isolated from A. stephensi midgut against selected class of antibiotics. [file 1471-2180-9-96-S1.doc]

**Additional File 1. Antibiotic sensitivity assay of microbial strains isolated from *Anopheles stephensi* midgut.**

| **S**  **No** | **Strain**  **No.** | **Antibiotics^a^** | | | | | | | | | | | | |
| --- | --- | --- | --- | --- | --- | --- | --- | --- | --- | --- | --- | --- | --- | --- |
|  |  | Amp | Car | Chl | Gen | Kan | Nal | Peni | | Poly | | Rif | Str | Tet |
|  |  | 25  mcg | 100  mcg | 10  mcg | 10  mcg | 30  mcg | 30  mcg | 10  units | | 100  units | | 15  mcg | 10  mcg | 10  mcg |
|  |  | Zone of inhibition (mm) | | | | | | | | | | | | |
| **Field-collected *A. stephensi* females** | | | | | | | | | | | | | | |
|  | F1 | 7 | 10 | 20 | 20 | 35 | 13 | | 7 | | 7 | 8 | 37 | 13 |
|  | F2 | 7 | 13 | 22 | 11 | 14 | 17 | | 7 | | 9 | 8 | 16 | 16 |
|  | F3 | 15 | 27 | 17 | 17 | 15 | 20 | | 8 | | 9 | 9 | 18 | 14 |
|  | F4 | 17 | 26 | 17 | 22 | 22 | 20 | | 7 | | 11 | 22 | 25 | 30 |
|  | F5 | 14 | 16 | 20 | 23 | 25 | 11 | | 7 | | 7 | 8 | 34 | 16 |
|  | F6 | 16 | 20 | 14 | 15 | 18 | 19 | | 7 | | 11 | 7 | 18 | 22 |
|  | F8 | 21 | 24 | 12 | 20 | 21 | 22 | | 9 | | 12 | 20 | 21 | 30 |
|  | F10 | 35 | 35 | 30 | 20 | 21 | 34 | | 22 | | 15 | 27 | 20 | 30 |
|  | F11 | 20 | 24 | 17 | 18 | 20 | 22 | | 10 | | 12 | 22 | 23 | 30 |
|  | F12 | 7 | 20 | 7 | 7 | 10 | 15 | | 7 | | 7 | 8 | 23 | 15 |
| **Field-collected *A. stephensi* Male** | | | | | | | | | | | | | | |
|  | M2 | 8 | 10 | 22 | 22 | 23 | 24 | | 7 | | 14 | 24 | 18 | 25 |
|  | M3 | 10 | 20 | 15 | 20 | 16 | 16 | | 7 | | 10 | 15 | 17 | 22 |
|  | M5 | 10 | 16 | 11 | 17 | 20 | 14 | | 7 | | 9 | 14 | 13 | 23 |
|  | M6 | 19 | 30 | 26 | 20 | 15 | 20 | | 9 | | 11 | 11 | 18 | 17 |
|  | M7 | 20 | 24 | 25 | 23 | 19 | 22 | | 12 | | 10 | 21 | 18 | 23 |
|  | M8 | 18 | 20 | 18 | 20 | 14 | 10 | | 22 | | 8 | 32 | 11 | 26 |
|  | M9 | 10 | 16 | 18 | 13 | 16 | 14 | | 8 | | 10 | 15 | 21 | 20 |
|  | M10 | 30 | 32 | 25 | 22 | 20 | 7 | | 19 | | 8 | 30 | 21 | 20 |
|  | M11 | 15 | 24 | 25 | 24 | 20 | 17 | | 15 | | 10 | 23 | 21 | 25 |
|  | M13 | 11 | 16 | 12 | 20 | 16 | 12 | | 7 | | 11 | 16 | 14 | 22 |
|  | M15 | 18 | 18 | 22 | 20 | 16 | 21 | | 9 | | 14 | 11 | 20 | 18 |
|  | M16 | 10 | 17 | 20 | 15 | 16 | 9 | | 7 | | 11 | 14 | 20 | 22 |
| **Field-collected *A. stephensi* larvae** | | | | | | | | | | | | | | |
|  | L1 | 38 | 36 | 18 | 16 | 15 | 10 | | 29 | | 8 | 22 | 7 | 23 |
|  | L2 | 9 | 8 | 17 | 16 | 11 | 13 | | 8 | | 7 | 11 | 16 | 18 |
|  | L3 | 29 | 30 | 25 | 25 | 25 | 22 | | 35 | | 15 | 40 | 7 | 35 |
|  | L8 | 38 | 40 | 18 | 16 | 7 | 10 | | 15 | | 9 | 25 | 7 | 27 |
|  | L5 | 31 | 30 | 18 | 11 | 12 | 10 | | 25 | | 9 | 22 | 7 | 23 |
|  | L7 | 26 | 30 | 15 | 7 | 8 | 7 | | 23 | | 7 | 17 | 8 | 10 |

a: Ampicillin (Amp), Carbenicillin (Car), Chloramphenicol (Chl), Gentamycin (Gen), Kanamycin (Kan), Nalidixic acid (Nal), Penicillin G (Peni), Polymyxin B (Poly), Rifampicin (Rif), Streptomycin (Str) and Tetracyclin (Tet)
